# Maternal obesity and the impact of associated early-life inflammation on long-term health of offspring

**DOI:** 10.3389/fcimb.2022.940937

**Published:** 2022-09-16

**Authors:** Merve Denizli, Maegan L. Capitano, Kok Lim Kua

**Affiliations:** ^1^ Department of Pediatrics, Division of Neonatal-Perinatal Medicine, Indiana University School of Medicine, Indianapolis IN, United States; ^2^ Department of Microbiology & Immunology, Indiana University School of Medicine, Indianapolis IN, United States

**Keywords:** maternal obesity, maternal inflammation during pregnancy, offspring of mothers with obesity, developmental programming of adult diseases, long-term health of offspring in obese mothers

## Abstract

The prevalence of obesity is increasingly common in the United States, with ~25% of women of reproductive age being overweight or obese. Metaflammation, a chronic low grade inflammatory state caused by altered metabolism, is often present in pregnancies complicated by obesity. As a result, the fetuses of mothers who are obese are exposed to an in-utero environment that has altered nutrients and cytokines. Notably, both human and preclinical studies have shown that children born to mothers with obesity have higher risks of developing chronic illnesses affecting various organ systems. In this review, the authors sought to present the role of cytokines and inflammation during healthy pregnancy and determine how maternal obesity changes the inflammatory landscape of the mother, leading to fetal reprogramming. Next, the negative long-term impact on offspring’s health in numerous disease contexts, including offspring’s risk of developing neuropsychiatric disorders (autism, attention deficit and hyperactive disorder), metabolic diseases (obesity, type 2 diabetes), atopy, and malignancies will be discussed along with the potential of altered immune/inflammatory status in offspring as a contributor of these diseases. Finally, the authors will list critical knowledge gaps in the field of developmental programming of health and diseases in the context of offspring of mothers with obesity, particularly the understudied role of hematopoietic stem and progenitor cells.

## Introduction

The prevalence of obesity in women of childbearing age has been steadily increasing for the past three decades (Poston et al., 2016; Chen et al., 2018). In the United States, the prevalence of obesity among women of childbearing age was up to 40% in 2017-2018 (Hales et al., 2020). Maternal obesity is associated with poor outcomes in both mothers and their offspring. Mothers with pre-pregnancy obesity are more likely to have infertility, spontaneous pregnancy loss, congenital anomalies, gestational diabetes, higher risk of cesarean delivery, wound complications, increased risk of venous thromboembolism, depression, and difficulty breastfeeding (Sebire et al., 2001; Rasmussen, 2007; Luke et al., 2011; Wloch et al., 2012; Cnattingius et al., 2013; Aune et al., 2014; Gyamf, 2014; Tuthill et al., 2022). In addition to the health concerns for mothers with obesity, children born to mothers with obesity are more likely to suffer from numerous chronic illnesses throughout their life. These illnesses include obesity, cardiovascular complications, and neuropsychiatric disorders (Getz et al., 2016; Andersen et al., 2018; Voerman et al., 2019; Razaz et al., 2020). In fact, both pre-clinical and clinical studies support the conclusion that exposure to a suboptimal in-utero environment predisposes offspring to developing these chronic conditions. However, our knowledge regarding underlying mechanisms of this programming remains limited. “Metaflammation” is a low-grade inflammatory state secondary to an impaired immune cell profile that leads to activation of pro-inflammatory pathways and is increasingly recognized as an early life factor that shapes offspring health (Gregor and Hotamisligil, 2011; Pantham et al., 2015). Although pregnancy itself is characterized by an altered inflammatory profile compared to the non-pregnant state, a tightly regulated balance between pro- and anti-inflammatory cytokines is necessary for implantation, placentation and continuation of a healthy pregnancy (Ashkar et al., 2000; Aluvihare et al., 2004; Fest et al., 2007; Care et al., 2013; Griffith et al., 2017). Maternal obesity is associated with a chronic metabolic inflammatory state that skews this tight balance toward a pro-inflammatory state (Sisino et al., 2013; Thakali et al., 2014; Saben et al., 2014; Nakajima et al., 2016; Castellana et al., 2018). In this review, we will summarize the common models used to study the immune system during normal pregnancy and pregnancy complicated by maternal obesity, and briefly discuss the role of inflammation during healthy pregnancy and known changes associated with maternal obesity. In the latter part of this review, we will provide an in-depth review of the adverse effects of maternal obesity on offspring long-term health and propose a new model where the altered immune function in offspring potentially contributes to different disease states.

## Models of pregnancy and maternal obesity

Clinical studies designed to obtain longitudinal data and biosamples over different stages of pregnancy from mothers with normal weight (BMI 20-25) and mothers with obesity (BMI>30) are undoubtedly a powerful tool (Haas et al., 2015; Rees et al., 2022). In fact, they provide a comprehensive understanding, defining the changes in circulating cytokines and immune cell profiles associated with maternal obesity (Maguire et al., 2021; Sureshchandra et al., 2021a; Ross et al., 2022). Cord blood and placental tissues collected at delivery also facilitate the understanding of immune adaptations of different compartments (maternal plasma, maternal-fetal interface, fetal plasma) during pregnancy (Challier et al., 2008; Laskewitz et al., 2019; Enninga et al., 2021; Jaramillo-Ospina et al., 2021). While such studies provide a direct representation of the biology of pregnancy in humans, it is not known if the findings are directly due to impact of maternal obesity or a consequence of other factors contributing to development of obesity, such as unhealthy diet or genetic risks. Further, it is not possible to evaluate longitudinal changes in cytokines and immune cell profiles at maternal-fetal interface and fetal circulations. Finally, human epidemiology studies demonstrate the associations of maternal obesity exposure and adverse health in offspring, but these studies do not inform the direct contributions of maternal obesity and are not designed to inform the molecular mechanisms contributing to offspring disease development.

Animal models are often used to overcome the limitations of human studies. Maternal obesity has been modelled using murine (Lee et al., 2020; Mu et al., 2022; Zhang et al., 2022; Thapa et al., 2022), rat (Lin et al., 2019; Zhang et al., 2020; Deshpande et al., 2021), and non-human primate models (Rivera et al., 2015; Harris et al., 2016; Salati et al., 2019). Murine and rat models are most used to discern the effects and mechanisms of transgenerational disease transmission due to their short gestation duration. Murine model systems also allow researchers to dissect the effects of specific maternal factors (e.g., overnutrition, obesity, insulin resistance) on offspring disease development, and delineate molecular pathways in different tissues and organ systems of offspring. The main strategy used to induce pre-pregnancy obesity in murine and rat models is by using maternal high-fat (Lin et al., 2019; Moazzam et al., 2021; Zhou et al., 2021) or Western style (high-fat high-sucrose) diet (Muller et al., 1985; Victorio et al., 2021; Chung et al., 2021; Chaix et al., 2021). A major limitation of such an approach is that the fetal and newborn weight are often lower in pups born to obese dams that were fed high-fat diet (Christians et al., 2019). However, the use of Western diet with 45% fat calories appears to produce neonatal pups with unchanged (Fornes et al., 2017) or higher (Rosario et al., 2015; Aye et al., 2015) birthweight. Such an approach may be more representative of pregnancy complicated by maternal obesity, but still does not differentiate the contribution of the diet vs. maternal adiposity. This limitation can be circumvented with different strategies. For example, pregnant animals can be fed high-fat at selected window (Cerf and Louw, 2014) and newborn pups can be cross-fostered to discern the role of high-fat diet at different period of gestation and lactation. Alternatively, Isganaitis et al. have used the haploinsufficient insulin receptor substrate-1 (IRS-1) mice to model maternal obesity and maternal insulin resistance during pregnancy without the need of diet-induction (Isganaitis et al., 2014). Additionally, researchers must be cognizant of key differences in immune cell development of murine models and humans. For example, the liver and spleen are found to be extramedullary hematopoietic sites in neonatal mice (Wolber et al., 2002). On the other hand, the development of immune systems in non-human primates closely resembles that of humans (Makori et al., 2003). Therefore, non-human primates are a suitable model to understand the immune adaptations of the maternal-fetal interface and fetal circulations, as well as how this adaption responds to maternal obesity (Salati et al., 2019; Dunn et al., 2022).

## Inflammation in pregnancy

**Figure 1:**
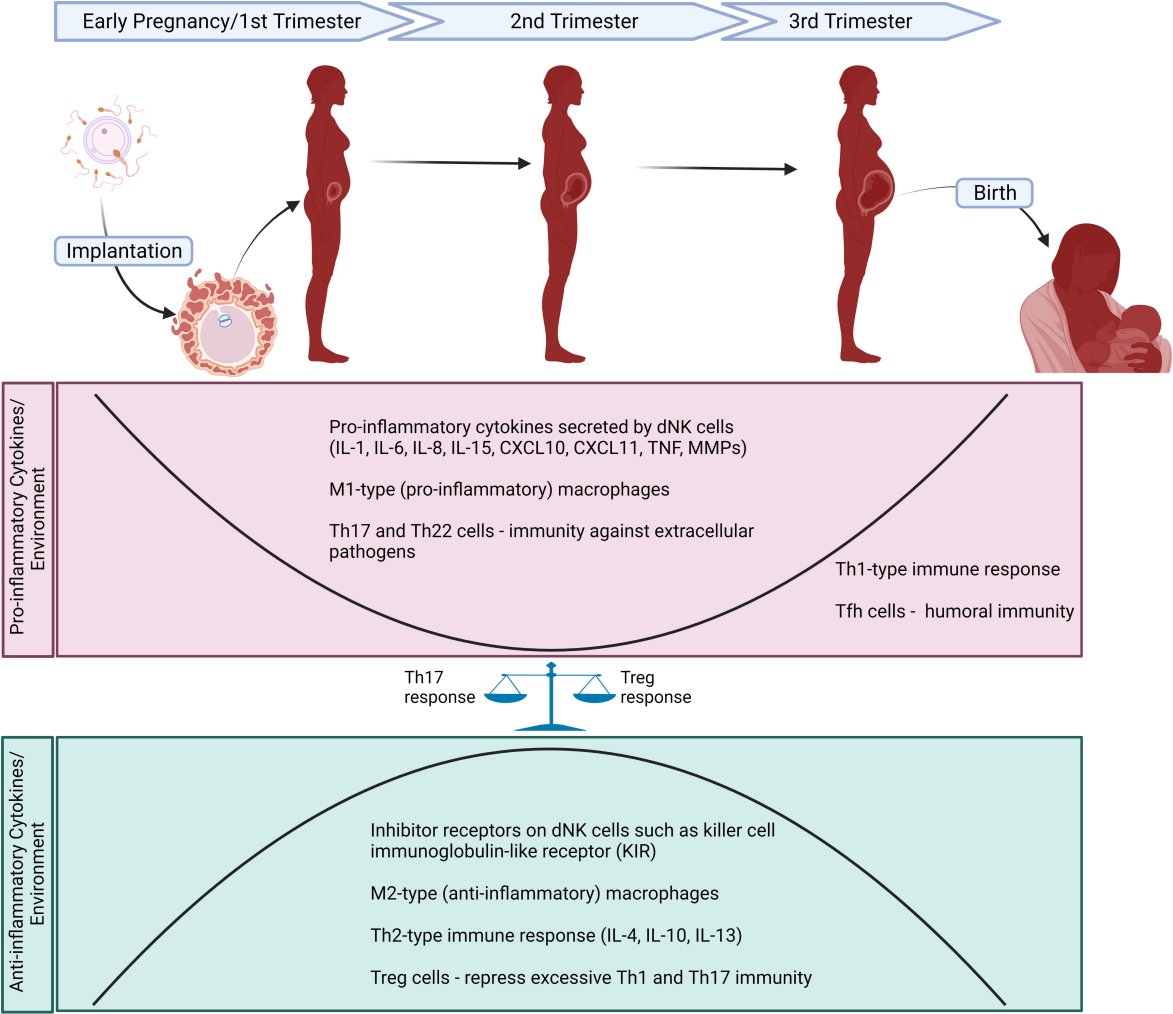
Schematic diagram of major changes in immune cells and inflammatory markers/cytokines during different stages of a normal pregnancy. First trimester is characterized by an initial pro-inflammatory stage necessary for implantation and placentation. dNK cells secrete high levels of pro-inflammatory factors including IL-1, IL-6, IL-8, IL-15, CXCL10, CXCL11, TNF, MMPs. Predominance of M1-type (pro-inflammatory) macrophages contribute to the inflammatory state observed in first trimester. At second trimester, Th2-type immune response and immunomodulatory cytokines secreted by Th2 cells (e.g., IL-4, IL-10, IL-13) lead to an anti-inflammatory stage that is crucial for fetal growth. M2-type (anti-inflammatory) macrophages and increased expression of inhibitory receptors on dNK cells such as killer cell immunoglobulin-like receptor (KIR) play a significant role in maintaining immune tolerance in maternal-fetal interface. Finally, third trimester is defined as a second pro-inflammatory stage and Th1-type immune state is thought to initiate labor and delivery. Other T helper cells including Th17, Th22, Treg and Tfh cells are also essential for healthy pregnancy. TH17 and Th22 cells offer immunity against extracellular pathogens at the maternal-fetal interface. Treg cells increase immune tolerance to fetus by repressing uncontrolled Th1 and Th17 immunity. Tfh cells provide humoral immunity by priming B cells to initiate extrafollicular and germinal center antibody responses in third trimester. Created with BioRender.com.

Strict regulation of inflammatory factors is required for implantation, placentation, and continuation of pregnancy (Gude et al., 2004; Mor et al., 2017). The maternal body’s physiological adaptation to pregnancy is determined by controlled production of cytokines and other inflammatory factors by diverse cell subtypes within the maternal-fetal interface (**Figure 1**) (Abrahams et al., 2004; Mjosberg et al., 2010; Svensson et al., 2011; Amsalem et al., 2014). Changes in immune cell types in the placenta including lymphocytes, natural killer (NK) cells, neutrophils, dendritic cells, and innate lymphoid cells during different stages of pregnancy are explained in detail by St-Germain et al. (2020). In general, the immune regulation of a pregnant mother is controlled by adaptive T helper cells (Huang et al., 2020) and innate immune responses (Aghaeepour et al., 2017). However, a majority of these studies are based on circulating cytokines and immune cell profiles. Type 1 T helper (Th1) and type 2 T helper (Th2) lymphocytes are two major subsets of CD4^+^ T helper (Th) cells that regulate the adaptive immune response (Zhu, 2018). Th1 cells produce high levels of interferon (IFN)-γ, interleukin (IL)-2 and tumor necrosis factor (TNF)-beta and are responsible for phagocyte-dependent inflammation, as well as protection against intracellular pathogens (Zhu, 2018). They also play an important role in the development of organ-specific autoimmune diseases and chronic inflammatory disorders (Zhu, 2018). Th2 cells produce IL-4, IL-5, IL-6, IL-9, IL-10, and IL-13, which leads to strong antibody responses by regulation of the class switch recombination of B cells and eosinophil activation but inhibit phagocytic cell function (Romagnani, 2000). Mor et al. described three distinct immunologic stages based on the body’s inflammatory response during pregnancy (Mor et al., 2017). During the first trimester, there is an initial pro-inflammatory stage that is vital for implantation and placentation. As the second trimester begins, there is an anti-inflammatory and Th2-type immune stage that is necessary for fetal growth. Finally at the third trimester, a second pro-inflammatory stage and Th1-type immune state is thought to initiate labor and delivery (Mor et al., 2017). In addition to the essential role of Th1 and Th2 cells during pregnancy, other T helper cells, such as T helper 17 (Th17), T helper 22 (Th22), follicular T helper (Tfh) and regulatory T cells (Treg) in the maternal-fetal interface contribute to continuation of a healthy pregnancy. Th17 and Th22 cells are involved in the induction of immunity against extracellular pathogens at the maternal-fetal interface (Bettelli et al., 2006; Barnes et al., 2021). Uncontrolled Th1 and Th17 response is associated with implantation failure and pregnancy loss (Kwak-Kim et al., 2003; Wang et al., 2010; Lee et al., 2012). Treg cells enhance immune tolerance to fetus by repressing excessive Th1 and Th17 immunity and autoimmune response (Aluvihare et al., 2004). Tfh cells are enriched in the third trimester and offer humoral immunity as they are known to prime B cells to initiate extrafollicular and germinal center antibody responses (Monteiro et al., 2017). Tfh cells also balance Th1/Th2 immunity by favoring Th2 immunity. T helper cell profile in pregnancy is discussed in detail by Wang et al. (2020).

Other members of the innate immune system, such as NK cells, mast cells, macrophages, dendritic cells, and neutrophils play a key role in fine-tuning immunologic stages involved in pregnancy. First trimester human decidual leukocytes are primarily NK cells (70%) and macrophages (20%) (Moffett-King, 2002; Huhn et al., 2021). Decidual NK (dNK) cells express higher levels of chemokines, cytokines, and angiogenic factors compared to peripheral blood NK (pNK) cells. In early stages of pregnancy, dNK cells play a key role in implantation by secreting pro-inflammatory factors including IL-8, IL-15, IL-6, CXCL10 and CXCL11 (Zhang and Wei, 2021). The dNK cells are also involved in remodeling the endometrial vasculature by producing angiogenic factors such as vascular endothelial growth factor C (VEGFC), placental growth factor (PIGF) angiopoietin 2 (ANG2), IL-8, angiogenin, stromal-derived factor-1 (SDF-1/CXCL12), IFN-γ, matrix metalloproteinase (MMP)9 and MMP2 (Moffett-King, 2002; Radomska-Lesniewska et al., 2021). On the other hand, inhibitor receptors expressed highly on dNK cells, such as killer cell immunoglobulin-like receptor (KIR), recognize HLA ligands to inhibit NK cell cytotoxicity and maintain immune tolerance in maternal-fetal interface during the anti-inflammatory stage of pregnancy (Ferreira et al., 2017). At late gestation, reactivation of dNK cells and secretion of pro-inflammatory cytokines from activated dNK cells induce parturition by breaking immune tolerance (Zhang and Wei, 2021). Dysregulation of dNK cells is found to be associated with adverse pregnancy outcomes including recurrent spontaneous abortions, preeclampsia as well as other reproductive problems such as endometriosis and recurrent implantation failure (Fu et al., 2021; Fukui et al., 2021). Macrophages also play an important role in maintaining a healthy pregnancy. For instance, the predominance of M2 (anti-inflammatory or alternatively activated) type macrophages, as opposed to M1 (pro-inflammatory or classically activated) type macrophages, are necessary at the maternal-fetal interface to sustain fetomaternal tolerance for a healthy pregnancy (Yao et al., 2019). A recent study evaluated major immune cell subsets along with functions using mass cytometry. It showed that the regulation of the immune system in healthy term pregnancies is precisely timed and proposed the novel role of interleukin-2-dependent STAT5ab activation as modulatory pathway of this response (Aghaeepour et al., 2017).

## Maternal obesity and inflammation

Despite some inconsistent results, most studies in the literature have shown that mothers with pre-pregnancy obesity have elevated pro-inflammatory markers/cytokines, such as IL-8, IL-6, CRP, TNF-α and IFN-γ (Madan et al., 2009; Zhu et al., 2010; Englich et al., 2017; Kretschmer et al., 2020; Maguire et al., 2021) and altered adipokines (Hinkle et al., 2019; Jara et al., 2020; Jaramillo-Ospina et al., 2021). However, it is important to know that the changes in cytokines are inconsistent, as discussed by Pendeloski et al. (2017). The inconsistent results may be due to a variety of factors, including the biological variabilities within human populations, types of samples (serum vs. plasma), or fasting states of the mothers (de Jager et al., 2009; Lee et al., 2016; Martinez-Garcia et al., 2019). Nevertheless, there is still concern that an altered inflammatory state can negatively affect the growing fetus indirectly by altering a variety of placental functions (e.g., trophoblast invasion, nutrient transport) (Kwak-Kim et al., 2014; Goldstein et al., 2020). Aye et al. found that increased maternal body mass index (BMI) is associated with activation of placental p38-MAPK and STAT signaling without changes in the classical inflammatory pathways or fetal systemic inflammatory profile (Aye et al., 2014). This finding again demonstrates the difference in immune adaptions in response to stressors and suggests that inflammation associated with maternal obesity is regulated by altered placental function. Conversely, there is evidence that elevated maternal cytokines during immune activation secondary to infection increases cytokine levels in the fetal compartment in preterm infants of both humans (Shobokshi and Shaarawy, 2002) and the rhesus monkey (Short et al., 2010), highlighting the differences in immune response/adaptations to specific stressors in different maternal-fetal compartments. Nevertheless, these findings suggest that immune activation/pro-inflammatory state in the mother could result in increased levels of cytokines in the growing fetus, either by inducing cytokine secretion from the placenta or by direct transport across the placenta.

## Adverse effects of maternal obesity on offspring health

In a meta-analysis performed by Lutsiv et al, maternal obesity is associated with maternal and infant adverse outcomes including preeclampsia, gestational diabetes, Cesarean section, bleeding, low umbilical artery pH, low Apgar scores (appearance, pulse, grimace, activity, respiration), congenital birth defects and NICU (neonatal intensive care unit) admission (Lutsiv et al., 2015). In addition to its perinatal adverse outcomes, maternal obesity is also associated with long-term diseases in children. In a seminal article by Barker in 1990, Dr. David Barker proposed the model of “developmental origins of adult disease”. There, he hypothesized that the exposure to a suboptimal environment during early life (fetal and infant) shapes future health of an individual (Barker, 1990). He initially showed that adults born with low birthweight are at higher risks of developing metabolic and cardiovascular diseases, secondary to intrauterine growth restriction from insufficient nutrient intake. In contrast, conditions associated with in-utero “overnutrition” and increased inflammation, such as gestational diabetes and maternal obesity, also negatively impact offspring long-term health. Human data suggest that activated pro-inflammatory state during pregnancy is associated with long-term diseases of offspring including childhood obesity (Englich et al., 2017), neuropsychiatric disorders (Allswede et al., 2016; Ghassabian et al., 2018; Volk et al., 2020; Goldstein et al., 2021), and childhood wheezing/allergic diseases (Kim et al., 2008; Rothers et al., 2018). Complications seen with these diseases leave the children vulnerable for further complications such as suppressed immune response. For instance, childhood obesity is associated with poor response to immunizations (Simo Minana et al., 1996). While it is challenging to determine the underlying mechanism contributing to these diseases in human offspring, preclinical models implicated the role of increased inflammation during pregnancy complicated by maternal obesity. In a study performed with rats, offspring of diet-induced obese (DIO) dams had accelerated postnatal growth and higher total body adiposity (Howie et al., 2009). Interestingly, maternal supplementation with conjugated linoleic acid (CLA) during pregnancy, an anti-inflammatory lipid, led to reversal of the metabolic dysfunction in offspring of DIO dams. Further, maternal CLA supplementation also reversed the increase in TNF-α, IL-1β, and NLRP3 expression in the guts of male offspring from DIO dams, suggesting that these adverse effects seen from exposure to maternal obesity may arise from alterations in maternal inflammatory profiles (Reynolds et al., 2015). Further examination of the specific mechanisms responsible for sex-specific increase of pro-inflammatory cytokines in offspring of mothers with obesity is necessary.

### Neuropsychiatric disorders

There is growing evidence that children born to mothers with obesity are more prone to neurodevelopmental and neuropsychiatric disorders. For instance, a cohort analysis done in more than 240,000 deliveries showed that offspring of mothers with obesity had higher rates of autism spectrum disorder (ASD) and neuropsychiatric morbidity-related hospitalizations. Additional studies demonstrated that children born to mothers with obesity were found to have lower intelligence quotients (IQs) (Neggers et al., 2003; Tanda et al., 2013; Pugh et al., 2015; Coo et al., 2019), higher rates of ASD (Bilder et al., 2013; Gardner et al., 2015; Getz et al., 2016; Windham et al., 2019), attention deficit hyperactivity disorder (ADHD) (Buss et al., 2012; Andersen et al., 2018; Jenabi et al., 2019; Parker et al., 2022), cerebral palsy (Crisham Janik et al., 2013; Villamor et al., 2017; Xiao et al., 2018; Zhang et al., 2019) and affective disorders (Robinson et al., 2013; Mina et al., 2017). Animal models of maternal diet-induced obesity have been able to provide mechanistical insight as to why maternal obesity exposure led to neuropsychiatric morbidity in offspring. Significant alterations in brain structure have been noted in offspring of mothers with obesity in rodent models. These alterations include reduced proliferation of neural progenitors in the hippocampus (Tozuka et al., 2009), decreased apoptosis in hippocampus and neuronal differentiation in dentate gyrus (Niculescu and Lupu, 2009), impaired migration and maturation of neural stem cells in the ventricular regions and cortex (Stachowiak et al., 2013), dendritic atrophy in hippocampus and amygdala (Janthakhin et al., 2017), and decreased myelination in the offspring cortex (male offspring only) (Graf et al., 2016). It was also shown that offspring of DIO dams have impaired learning and cognition (White et al., 2009; Page et al., 2014; Ding et al., 2018; Mucellini et al., 2019), as well as behavioral abnormalities including hyperactivity (Dias et al., 2020), anxiety (Wright et al., 2011; Winther et al., 2018), decreased sociability (Kang et al., 2014; Buffington et al., 2016), disordered eating (Bayol et al., 2007; Vucetic et al., 2010) and addictive-like behaviors (Vucetic et al., 2010; Sarker et al., 2019), some of which were sex-specific. These behavioral phenotypes seen in rodents might be reflective of neuropsychiatric disorders including ADHD, anxiety disorder, ASD and schizophrenia. Increased lipid peroxidation, microglial activation, and increased pro-inflammatory cytokine expression in offspring of mothers with obesity suggest that neuroinflammation and oxidative stress play an important role in adverse neural outcomes. Microglia, resident macrophages that are derived from primitive myeloid precursor cells in extra-embryonal yolk sac and seed the brain rudiment during early fetal development, are good candidates for long-term changes in the brain due to their long lifespan and renewal ability (Alliot et al., 1999; Yoo and Kwon, 2021). Edlow et al. found that offspring of mice with obesity had an exaggerated TNF-α production in response to lipid polysaccharide (LPS) exposure in placental CD11b^+^ cells, as well as brain microglia compared to the control group (Edlow et al., 2019). Overproduction of pro-inflammatory cytokines was more prominent in male offspring, which may correlate with male predominance of certain neuropsychiatric morbidities associated with maternal obesity (Hanamsagar and Bilbo, 2016; Santos et al., 2022; Breach and Lenz, 2022). Similarly, another study demonstrated that offspring of DIO mothers had overactivation of microglial cells and increased toll-like receptor (TLR4) mRNA expression in the hippocampus and exaggerated hippocampal IL-1β responses to LPS challenge (Graf et al., 2016). The pro-inflammatory environment achieved by the increased expression of pro-inflammatory proteins pJNK and TNF-α affects brain derived neurotrophic factor (BDNF) metabolism and tryptophan hydroxylase 2 (TPH2) expression. BDNF is a crucial molecule for hippocampal neurogenesis and TPH2 is a key enzyme for serotonin synthesis. Changes to BDNF metabolism and TPH2 expression are both known to be associated with increased anxiety-like behavior in adulthood (Peleg-Raibstein et al., 2012; Dias et al., 2020). In addition to the rodent data, maternal high-fat diet and subsequent adiposity was found to be associated with an elevated number of microglia in the basolateral amygdala of juvenile non-human primate offspring (Dunn et al., 2022).

Although elucidating the molecular mechanisms involved in the sex-bias in neurodevelopmental disorders is an evolving topic, our knowledge as to why the incidence of neurodevelopmental disorders is higher in male offspring remains limited. One theory is that the sex-specific activation of immune pathways within the placenta may contribute to prenatal stress programming effects on the offspring. With a mouse model of early prenatal stress, Bronson and Bale showed that immune-related genes, including pro-inflammatory cytokines (IL-1β, IL-6) and chemokines (CCL5, chemokine ligand 10) were up-regulated by early prenatal stress (EPS), specifically in males, indicative of a pro-inflammatory state in placentas of male fetuses. The EPS effect was partially ameliorated by nonsteroidal anti-inflammatory drug (NSAID) treatment. Examination of male adult offspring revealed a hyperactive phenotype, which was reversed by maternal NSAID treatment in males. Males exposed to EPS were also found to have dopaminergic dysregulation, which may be the leading factor for hyperactive phenotype observed in EPS male offspring (Bronson and Bale, 2014). Specific sex chromosome genes may be involved in the vulnerability of the male placenta to maternal stress and inflammation and resultant susceptibility to neurodevelopmental disorders. X-linked gene O-linked N-acetylglucosamine transferase (OGT) levels are higher in the female placenta than the male placenta as it escapes X inactivation. OGT is known to increase the methylation of a global histone repressive mark - H3K27me3 (histone H3 trimethylated at lysine 27). Placental OGT levels are further decreased in both sexes in response to EPS, leading to significantly lower OGT levels in the male placenta. Significantly decreased levels of OGT and its control over H3K27me3 in the male placenta predisposes male chromatin to be in a reactive state and more vulnerable to maternal stress (Bale, 2016).

### Atopy and lung development

Several studies and meta-analyses reveal that children of mothers with obesity are at higher risk for developing atopic diseases, including atopic dermatitis and asthma (Harpsoe et al., 2013; Forno et al., 2014; Dumas et al., 2016; Polinski et al., 2017; Huang et al., 2018; Drucker et al., 2019; Liu et al., 2020; Wei et al., 2022). This increase in risk indicates that offspring born to mothers with obesity had altered function in immune checkpoints regulating the development atopy/allergy. Increased levels of maternal CRP and TNF-α, pro-inflammatory cytokines that are elevated in mothers with obesity, have been associated with wheezing and lower respiratory tract infections in offspring (Morales et al., 2011; Halonen et al., 2013). A recent study also linked higher cord leptin levels with higher asthma risk at 3 years old in children born to mothers with obesity (Castro-Rodriguez et al., 2020).

This is supported by animal studies that investigated the relationship between maternal high-fat diet and offspring atopy. MacDonald et al. showed that offspring of DIO dams had increased airway hyperreactivity with methacholine challenge and end-inflation technique compared to offspring of normal-fat diet-fed mothers (MacDonald et al., 2017). Higher bronchial alveolar lavage fluid cell count with an increased neutrophil percentage and elevated concentration of IL-6 was also observed in the same study suggesting that an activated pro-inflammatory state may play a role in reactive airway disease in children born to mothers with obesity. Another study found a female offspring-specific increase in methacholine reactivity, as well as elevated levels of pro-inflammatory cytokines (IL-1β, IL-5, and CXCL1), MMP-3 and MMP-8 in lung lavage (Pascoe et al., 2022). The exacerbated allergic response in offspring of obese mice was also observed in response to allergen (ovalbumin) challenge (RR et al., 2019). In this study, the increased response to ovalbumin challenge is potentially triggered by a higher peripheral blood mononuclear cells miR-155 that can stimulate Th2 response and a lower tracheal and lung tissue miR-133b that can induce higher TGF-β1 levels in lung lavage (RR et al., 2019). Offspring born to high-fat diet-fed mothers also had increased viral replication in their lung tissues when they were exposed to Respiratory syncytial virus (RSV) (Griffiths et al., 2016). In addition to the higher incidence of reactive airway disease, maternal obesity impacts fetal lung development and function (Mayor et al., 2015; Baack et al., 2016; Smoothy et al., 2019; Heyob et al., 2019). Although data highlights that maternal obesity is a risk factor for several pulmonary complications, molecular mechanisms underlying this association needs further investigation.

### Cardiometabolic diseases

Fetal exposure to metabolic derangements caused by maternal obesity predisposes children to cardiometabolic diseases by altering the development of key organs in cardiometabolic health. Large population studies have established that even at a young age (1-25 years), offspring born to mothers with obesity are at higher risk of developing cardiovascular diseases (excluding congenital heart disease) (Razaz et al., 2020). A recent meta-analysis also demonstrates the positive association between maternal pre-pregnancy BMI and offspring blood pressure, independent of offspring BMI (Eriksson et al., 2014; Eitmann et al., 2022). In addition to cardiovascular diseases, offspring born to mothers with obesity are also at a higher risk of developing a higher BMI, insulin resistance, and ultimately type 2 diabetes. An individual participant data meta-analysis among 162,129 mothers and their children from 37 pregnancies and birth cohorts from Europe, North America, and Australia suggested that higher maternal pre-pregnancy BMI and gestational weight gain were associated with an increased risk of offspring overweight/obesity throughout childhood, with the strongest effects at later ages (Voerman et al., 2019). The association of excessive maternal weight gain with later offspring BMI may be driven by both familial (genetic) risk and intra-uterine exposure risk. Indeed, a population study using a prospectively enrolled cohort showed maternal weight gain during pregnancy had stronger association in BMI of siblings born to mothers with normal BMI, suggesting the contribution of genetic risks. In contrast, in women with pre-pregnancy obesity, greater maternal weight gain had a stronger association with the BMI of unrelated children, suggesting the greater contributions of intrauterine mechanisms in offspring born to mothers with obesity (Godfrey et al., 2017). Literature suggests that children exposed to maternal obesity are at increased risk for developing metabolic syndrome, even if the mothers do not meet criteria for gestational diabetes mellitus (GDM) (Boney et al., 2005). Although association of excessive maternal weight gain with later offspring BMI is driven largely by shared familial risk factors for BMI, a sibling study in a prospective cohort showed that in women who are obese in early pregnancy, greater maternal weight gain may be associated with greater later offspring BMI *via* intrauterine mechanisms in addition to shared familial characteristics (Lawlor et al., 2011). Animal studies performed with diet-induced maternal obesity models support human data and tend to elucidate the mechanisms involved in the association of maternal obesity and offspring cardiometabolic disease. In a diet-induced maternal obesity murine model, Samuelsson et al. showed that offspring exposed to maternal obesity had increased adiposity, impaired lipid and glucose metabolism and higher blood pressures months after birth (Samuelsson et al., 2008). Several studies showed that maternal obesity induces sex-specific effects on glucose metabolism and the cardiometabolic profiles of offspring in favor of male sex (Sun et al., 2012; Lecoutre et al., 2016; Kulhanek et al., 2020; Casasnovas et al., 2021). One theory is that the sex-differences in pancreatic β-cell function may be partially due to increased oxidative stress in male islets (Yokomizo et al., 2014). The mRNA levels of NOX4 and NAD(P)H oxidase gp91phox, key regulators of superoxide production in isolated islets, were found to be increased in male offspring exposed to maternal high-fat diet, although there was no significant change in the mRNA levels of female offspring exposed to maternal high-fat diet. Estrogen in female offspring may play a protective role against oxidative stress caused by exposure to maternal obesity (Hernandez et al., 2000). Taken together, both human and animal studies demonstrate that maternal obesity is a risk factor for adverse cardiometabolic outcomes in offspring and male predominance of impaired insulin metabolism might be secondary to increased oxidative stress in pancreatic β-cells in the setting of estrogen scantness.

Another potential mechanism being investigated regarding the association between maternal obesity and dysregulated metabolic state of offspring is alterations in adipokine signaling secondary to increased adiposity. Adipokines are cytokines secreted by adipose tissue that regulate obesity-related low-grade state of inflammation. Some of the well-defined adipokines include leptin, adiponectin, resistin, and ghrelin. Leptin is the product of the obesity (ob) gene (Zhang et al., 1994). It is secreted into the blood by adipocytes and regulates appetite, metabolism and energy homeostasis through its specific receptor leptin receptor (LEPR) located in the ventromedial hypothalamic nucleus, dorsomedial hypothalamic nucleus, lateral hypothalamic nucleus and arcuate nucleus. Insulin is a key regulator in leptin metabolism. Hyperinsulinemia leads to an increase in leptin concentration (Saad et al., 1998). Moreover, obesity is associated with a state of hyperleptinemia and decreased response to leptin which subsequently hinder leptin’s function leading to a dysregulation of energy homeostasis. Concentrations of leptin in cord blood of infants born to mothers with obesity was elevated compared to that of lean mothers (Guzman-Barcenas et al., 2016). These infants were also more insulin resistant that showed positive correlation with neonatal body fat (Catalano et al., 2009; Guzman-Barcenas et al., 2016; Costa et al., 2016). Animal studies support the human data (Rajia et al., 2010). High fat diet induced maternal obesity in mice was associated with hyperleptinemia, hyperinsulinemia, and hyperphagia in offspring and was exaggerated by postweaning high-fat diet (Rajia et al., 2010). Despite the clear evidence of elevated leptin levels in infants born to mothers with obesity, underlying mechanisms of the association between the increased leptin and being large for gestational age (LGA) or childhood obesity and whether there is evidence for inflammation of adipose tissue in these infants needs further investigation. In addition to its key regulatory function in energy homeostasis, leptin has emerged as an essential placental hormone that effects placental function, embryo implantation and fetal growth (Masuzaki et al., 1997; Ben et al., 2001; Magarinos et al., 2007; Perez-Perez et al., 2008; Childs et al., 2021). Various human studies demonstrated altered leptin metabolism in the placenta of mothers with obesity (Misra et al., 2013; Tuersunjiang et al., 2017). Maternal high-carbohydrate diet is associated with diminished leptin methylation in the placenta (Daniels et al., 2020). The impacts of altered placental leptin level/function in offspring of mothers with obesity are a complex process and differ in timing across pregnancy (Hinkle et al., 2019). Increased placental nutrient delivery secondary to increased leptin levels contributes to fetal overgrowth in offspring of mothers with obesity (Jansson et al., 2003).

### Altered gut microbiome

Novel studies suggest that gut microbiota differs between obese and lean individuals and plays a role in human obesity and associated metabolic risks. Considering that the first microbial exposure of offspring is the maternal microbiota during pregnancy, it can be speculated that the gut microbiome and the subsequent metabolic and immunological programming are influenced by maternal nutritional status. In a cohort study performed on 170 pregnant women, infants born to overweight or obese mothers had a lower abundance of short chain fatty acid producing bacteria and lower fecal butyric acid levels at 1 month of age, which may contribute to predicting the risk of elevated adiposity later in life (Gilley et al., 2022). Germ-free mice colonized with stool microbes from 2-week-old human infants born to mothers with obesity had increased intestinal permeability, impaired macrophage activity and increased periportal inflammation compared with those colonized with stool microbes from infants born to mothers with normal weight. Moreover, these mice showed accelerated weight gain and development of fatty liver following exposure to Western diet (Soderborg et al., 2018). Myles et al. showed that offspring of mice fed with Western diet had increased susceptibility to infection and its complications, higher incidence of experimental autoimmune encephalitis and anaphylaxis. Altered immune responses and disease susceptibility of offspring fed with Western diet was found to be associated with altered gut microbiota and cohousing the offspring of Western diet-fed mice and low-fat diet-fed mice to equilibrate their microbiomes rescued their susceptibility to infection (Myles et al., 2013). Increased risk of necrotizing enterocolitis following exposure to maternal obesity supports the increased susceptibility to infection secondary to altered gut microbiota (Babu et al., 2018). Altered gut microbiome in pups of mice fed with a high-fat diet was associated with increased susceptibility to Dextran sulfate sodium-induced colitis in adulthood and exacerbated expression of pro-inflammatory cytokines (Xie et al., 2018). Altered early microbiota is also associated with increased adiposity and increased risk of later obesity (Dogra et al., 2015). Pups from the recipients of high-fat diet fed mice gut microbiota showed increased cognitive and social-behavioral disorders (Liu et al., 2021). Like the other cohousing studies, cohousing these pups with pups from recipient of high-fat/high-fiber diet-fed mice gut microbiota showed improved cognition, sociability and a greater preference for social novelty (Liu et al., 2021). Another study performed on Yucatan pigs showed that maternal Western diet during gestation and lactation, even in the absence of obesity, led to increased blood triglycerides and free fatty acids as well as decreased gut microbiota activity in offspring (Val-Laillet et al., 2017). However, unlike rodents, piglets born to mothers fed with Western diet had better cognition with higher working memory and reference memory despite smaller hippocampal granular cell layer and decreased neurogenesis (Val-Laillet et al., 2017). Although data suggest that exposure to maternal obesity results in changes in gut microbiome, further studies to delineate the long-term effects of altered gut microbiota on offspring health and uncover underlying molecular mechanisms are needed.

### Leukemia/cancer

Little is known about the impact of maternal obesity on offspring tumorigenesis. In a prospective cohort study done in Pennsylvania, children born to mothers with pre-pregnancy BMIs of 40 or greater were found to have 57% higher leukemia risk (Stacy et al., 2019). Similarly, in a retrospective case-control study in California, children born to overweight mothers (BMI 25-30) were at increased risk of leukemia and excessive gestational weight gain was associated with increased risk of offspring astrocytoma (Contreras et al., 2016). Considering the significant role of early-life exposures to environmental stressors on offspring health, it is very likely that maternal obesity may increase offspring risks of developing cancer. This could occur indirectly, as offspring of mothers with obesity are more likely to become obese themselves, and obesity is an independent risk factor for malignancies (Divella et al., 2022). On the contrary, exposure to maternal obesity during early life may directly reprogram tissues of different organ systems such that they are more likely to undergo tumorigenesis. Animal studies also suggest that maternal obesity increases the risk of hepatocellular carcinoma (Sun et al., 2020), mammary cancer (La Merrill et al., 2010; Zhang et al., 2020), prostate cancer (Benesh et al., 2013; Yang et al., 2018; Liu et al., 2019) and lung cancer (Shi et al., 2021) and some of these increased risks are attributed to the reprogrammed immune cells in the tumor microenvironment. However, clinical significance of the data is understudied and needs further elucidation.

## Hematopoietic stem and progenitor cells as the cellular origin of altered immune function in offspring born to mothers with obesity

There is compelling evidence that exposure to maternal obesity and chronic inflammatory state in mothers increases offspring risk to developing a wide range of chronic diseases, many of which have features of altered immune/inflammatory activation (Kim et al., 2008; Allswede et al., 2016; Englich et al., 2017; Ghassabian et al., 2018; Rothers et al., 2018; Volk et al., 2020). Such findings warrant the investigation of the cellular origin of the altered immune states. All the immune cells originate from hematopoietic stem and progenitor cells (HSPC). The characterization of progenitor populations downstream of hematopoietic stem cells HSCs in the 2000s lead to a model where hematopoiesis is illustrated as a branching tree that originates from HSCs and finally branches down to mature blood cells (Orkin, 2000). Briefly, HSCs have hematopoietic stem cells (HSCs) have two fundamental characteristics: the ability to self-renew and differentiate into all mature blood lineages. Once HSC differentiates to multipotent progenitor (MPP) cells, MPP cells further differentiate into two major lineages: common myeloid progenitor (CMP) and common lymphoid progenitor (CLP). CMP further differentiates into megakaryocyte-erythroid progenitor (MEP) and granulocyte/monocyte progenitor (GMP), ultimately differentiating to all mature blood cells except for lymphoid lineage: erythrocytes, platelets, monocytes, macrophages and granulocytes (neutrophils, eosinophils, basophils). CLP further differentiates into B cells, T cells and NK cells (Lim et al., 2013). Recent studies have suggested that hematopoietic differentiation is more complicated than this classical model of hematopoiesis as the HSC pool is functionally and molecularly heterogenous and HSCs have clonal expansion capacity (Stier et al., 2002; Dykstra et al., 2007; Sanjuan-Pla et al., 2013; Pietras et al., 2015). Furthermore, the new “continuum model of hematopoiesis” suggests that hematopoiesis is a continuous process which lacks the distinct punctuated phenotypic changes within the subpopulations (Velten et al., 2017; Laurenti and Gottgens, 2018).

**Figure 2:**
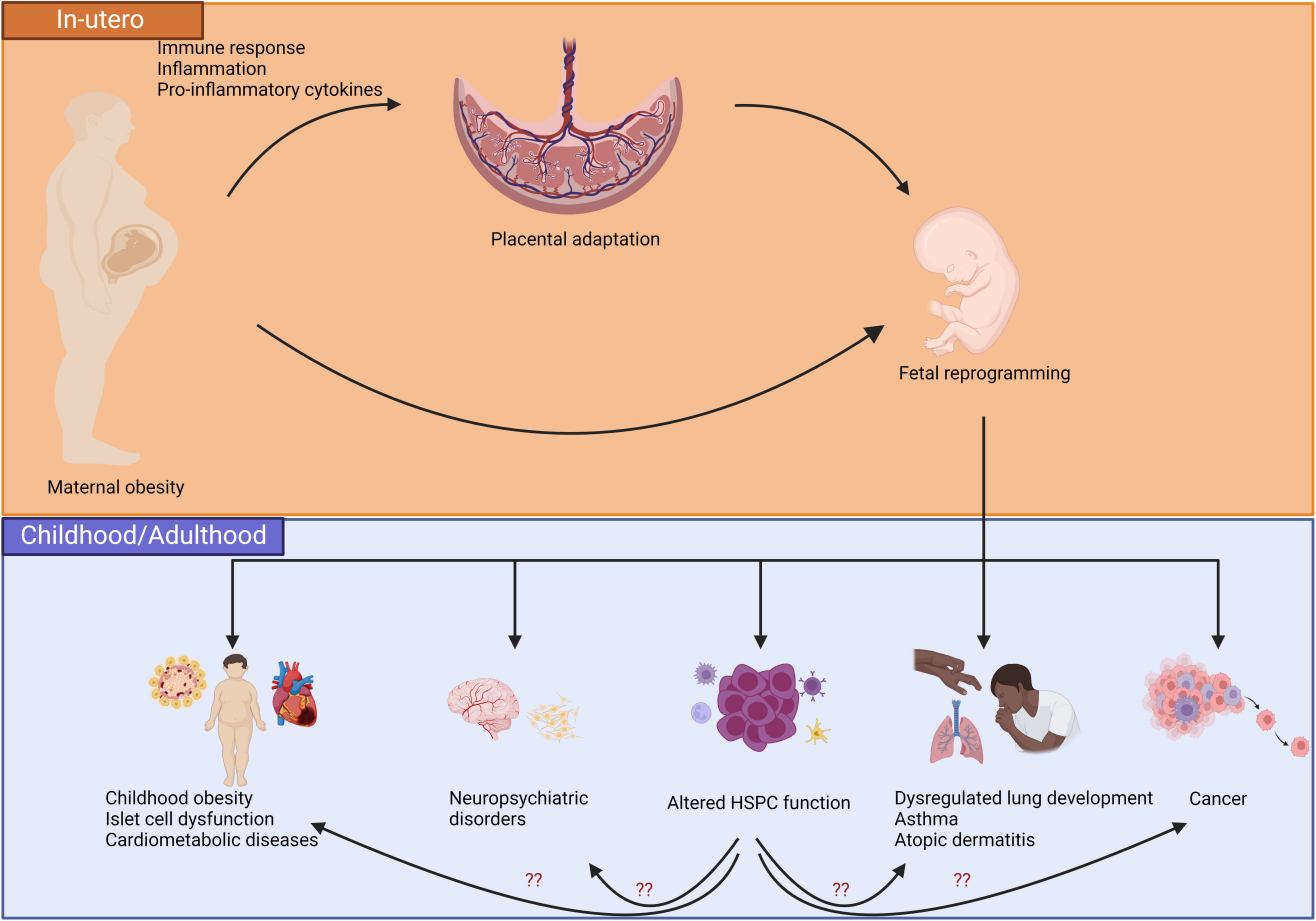
Proposed model of the effects of maternal obesity on placental adaptation and fetal reprogramming that led to end organ changes and adverse health outcomes in children. Placenta shows adaptation to environmental stressors including maternal obesity-associated inflammatory state. Changes in placenta predisposes the fetus to reprogramming that is associated with a higher risk of childhood obesity, neuropsychiatric disorders, altered HSPC function, atopic diseases, dysregulated pulmonary development, and/or cancer. Altered HSPC function and the immune cells that originate from the HSPCs may further predispose children to adverse health outcomes associated with maternal obesity. Created with BioRender.com.

Despite HSPC having to generate all functional hematopoietic lineages including immune cells, there are limited studies that focus on the impact of maternal obesity on hematopoiesis. Inflammation caused by several other factors is known to affect adult HSPC function (Masamoto et al., 2016; van den Berg et al., 2016; Emmons et al., 2017). However, the effects of the pro-inflammatory state during maternal obesity on long-term HSPC function in offspring has yet to be established. Numerous reviews have presented the evidence of altered fetal immunity in response to maternal obesity exposure (Sureshchandra et al., 2019; St-Germain et al., 2020; Monaco-Brown and Lawrence, 2022). In a study performed on a small cohort, cord blood mononuclear cells isolated from infants born to mothers with obesity showed an increased number of CD4^+^ cells and reduction in myeloid cell population. However, IL-12p40 and macrophage-derived chemokine, two molecules produced by activated myeloid cells and known to be chemoattractants for several other immune cells including macrophages, monocytes, Th2 cells, NK cells and dendritic cells, had increased concentration in infants born to mothers with obesity (Enninga et al., 2021). Another cohort study performed in 18 pregnant women showed that umbilical cord blood (UCB) collected from the placenta of mothers with obesity had increased lymphocyte subsets CD3^+^, CD4^+^, CD8^+^, NK, and CD8^+^CD25^+^Foxp3^+^ Treg cells while CD34^+^ cells, in which HSPCs are enriched, were decreased (Gonzalez-Espinosa et al., 2016). Moreover, exposure to maternal obesity was associated with an altered epigenome of CD4^+^ T cells in favor of effector memory cells, but with significant reduction in cytokine production in response to CD3/CD28 stimulation (Sureshchandra et al., 2021b). UCB monocytes collected from mothers with obesity had decreased response to LPS stimulation, which was found to be associated with hypomethylation within promoters and regulatory regions of genes involved in TLR-signaling in resting UCB monocytes (Sureshchandra et al., 2017). Pregnancies complicated by obesity were associated with reduced fetal monocyte and dendritic cell responses to TLR ligands (Wilson et al., 2015). The TLR family plays a key role in the pro-inflammatory response to bacterial infections; hence dysregulation of TLR signaling is associated with bacterial diseases including necrotizing enterocolitis (Jilling et al., 2006). Interestingly, Cifuentes-Zúñiga et al. found that leptin, a pro-inflammatory adipokine, was elevated in plasma of children born to mothers with obesity while monocytes and monocyte-derived macrophages of children exposed to maternal obesity revealed an anti-inflammatory phenotype, but with a suppression of anti-inflammatory mediators in response to an M2 polarization. The unbalanced response of monocytes to M1 and M2 stimulation in children of mothers with obesity might have detrimental effects on the inflammatory changes that could explain some of the chronic conditions associated with fetal re-programming caused by exposure to maternal obesity (Cifuentes-Zuniga et al., 2017). Again, the majority of these studies are performed using circulating immune cells and likely do not represent the landscape of immune cells in different organ systems as informed by animal studies [e.g immune cells within adipose tissue (Corken and Thakali, 2021) and liver (Nash et al., 2021)]. Nevertheless, these findings raise the possibility that the long-term function of HSPC is altered in offspring born to mothers with obesity. Currently, there is only one murine model that evaluated the acute effects of maternal high-fat diet on fetal HSPC function. Kamimae-Lanning investigated the impact of maternal obesity on fetal mice HSPC isolated from liver, which is the primary hematopoietic organ in-utero (Kamimae-Lanning et al., 2015). They reported that fetuses of female mice who were chronically fed a high-fat diet showed not only indications of adverse fetal programming including growth restriction, but also a reduction in HSPC in fetal livers. Despite the decrease in the total number of HSPC, the proportion of B220^+^ lymphoid and Gr-1^+^/Ter119^+^ myeloid cells in HFD livers were increased, suggesting a tendency towards myeloid and B cell differentiation. No significant difference was observed in the percentage of CD3^+^ cells. A competitive transplantation study performed by transplanting high-fat diet versus control diet-programmed fetal liver cells into the irradiated mice conditioned with either a high-fat diet or control diet showed that the chimerism of high fat diet-conditioned fetal liver cells was significantly compromised in male high-fat diet fed mice. Transcriptome analysis of high fat diet-programmed fetal livers revealed several transcriptionally altered targets that have roles in regulation of multiple pathways including development, metabolism, immunity and inflammation, complement activation, insulin signaling, and hematopoiesis. Although some of the studies performed on human and rodents demonstrate controversial results and the underlying mechanisms need further investigation, it is evident that exposure to maternal obesity has a momentous impact on immune response of the offspring (Wilson et al., 2015; Gonzalez-Espinosa et al., 2016; Enninga et al., 2021; Sureshchandra et al., 2021b). **Figure 2** proposes a new concept into the effects of maternal obesity on HSPC and potential inflammatory/immune pathways caused by altered HSPC function that may result in disorders associated with maternal obesity as mentioned above.

## Conclusion

Both human and animal studies demonstrate that offspring born to mothers with obesity are at higher risk of developing a wide range of chronic illnesses. However, current understanding of how different pathways are activated in the context of offspring of mothers with obesity, and the exact mechanisms leading to developmental programming, remains poorly defined. There is now strong evidence that early changes in inflammatory markers might be predictors of different morbidities later in life. Thus, evaluation of offspring inflammatory profiles of offspring at different stages of development needs further investigation. Additionally, there is a critical need to define the increasingly recognized sex-differences of disease susceptibility in offspring using both human epidemiological data and animal studies. Furthermore, the link between prenatal exposure to metabolic and inflammatory changes lead by maternal obesity and childhood/adult offspring diseases involving immune modulation reveals the necessity of better understanding the impact of metabolic dysregulation on HSPC.

## Author contributions

MD wrote the first draft of manuscript. MD, MC, and KK contributed in review of literature and editing the manuscript and approved the submitted version. All authors contributed to the article and approved the submitted version.

## Funding

The writing of this review was supported by funding from Indiana University School of Medicine, Indiana University Simon Cancer Center, March of Dimes Foundation (KK), and the U.S. Public Health Grant from the National Institutes of Health U54 DK106846 (MC) and K08HD109636 (KK).

## Acknowledgments

We thank Christina Santengelo (Indiana University Independent Investigator Incubator Program Scientific Writing mentor) in reviewing the manuscript. The authors wish to extend their apologies to our colleagues whose work we were unable to cite due to space limitations.

## Conflict of interest

The reviewers 'XH' and 'BG' declared past co-authorships with one of the authors 'MC' and the absence of any ongoing collaboration with any of the authors to the handling editor.

KK owns publicly traded stocks (TMO, DHR, DXCM).

The remaining authors declare that the research was conducted in the absence of any commercial or financial relationships that could be construed as a potential conflict of interest.

## Publisher’s note

All claims expressed in this article are solely those of the authors and do not necessarily represent those of their affiliated organizations, or those of the publisher, the editors and the reviewers. Any product that may be evaluated in this article, or claim that may be made by its manufacturer, is not guaranteed or endorsed by the publisher.
